# Impact of Omega-3 Fatty Acids Nano-Formulation on Growth, Antioxidant Potential, Fillet Quality, Immunity, Autophagy-Related Genes and *Aeromonas hydrophila* Resistance in Nile Tilapia (*Oreochromis niloticus*)

**DOI:** 10.3390/antiox11081523

**Published:** 2022-08-04

**Authors:** Doaa Ibrahim, Ahmed H. Arisha, Safaa I. Khater, Wafaa M. Gad, Zeinab Hassan, Sally H. Abou-Khadra, Dalia Ibrahim Mohamed, Tamer Ahmed Ismail, Sara A. Gad, Salwa A. M. Eid, Reham A. Abd El-Wahab, Asmaa T. Y. Kishawy

**Affiliations:** 1 Department of Nutrition and Clinical Nutrition, Faculty of Veterinary Medicine, Zagazig University, Zagazig 44511, Egypt; 2 Department of Animal Physiology and Biochemistry, Faculty of Veterinary Medicine, Badr University in Cairo (BUC), Badr City, Cairo 11829, Egypt; 3 Department of Physiology, Faculty of Veterinary Medicine, Zagazig University, Zagazig 44511, Egypt; 4 Department of Biochemistry, Faculty of Veterinary Medicine, Zagazig University, Zagazig 44511, Egypt; 5 Department of Bacteriology, Animal Health Research Institute (AHRI), Mansoura Branch Agriculture Research Center, Mansoura 35511, Egypt; 6 Fish Disease Department, Faculty of Veterinary Medicine, Aswan University, Aswan 81528, Egypt; 7 Microbiology Department, Zagazig Branch, Animal Health Research Institute (AHRI), Agriculture Research Center (ARC), Zagazig 44511, Egypt; 8 Department of Biochemistry, Zagazig Branch, Animal Health Research Institute (AHRI), Agriculture Research Center, Zagazig 44511, Egypt; 9 Department of Clinical Laboratory Sciences, Turabah University College, Taif University, P.O. Box 11099, Taif 21944, Saudi Arabia; 10 Department of Clinical Pathology, Animal Health Research Institute (AHRI), Zagazig Branch, Agriculture Research Center (ARC), Zagazig 44511, Egypt; 11 Biochemistry department, Animal Health Research Institute (AHRI), Mansoura Branch, Agriculture Research Center (ARC), 246 Dokki, Giza 12618, Egypt

**Keywords:** omega-3-PUFA, nano-formulation, performance, DHA/EPA, oxidative stress, autophagy, immune response, disease resistance

## Abstract

In modern aquaculture, enriching Nile tilapia’s diet with omega-3 poly-unsaturated fatty acids (PUFAs) not only plays an important role in its general health but also fortifies its fillet with omega-3-PUFAs. However, the major challenge affecting their delivery is their high instability due to oxidative deterioration. Thus, the prospective incorporation of omega-3-PUFAs into nanocarriers can enhance their stability and bioactivity. In this regard, the effect of reformulated omega-3-NPs was investigated on Nile tilapia’s performance, flesh antioxidant stability, immunity, and disease resistance. Four fish groups supplemented with omega-3-PUFAs-loaded nanoparticles (omega-3 NPs) at levels of 0, 1, 2, and 3 g/kg diet and at the end of feeding trial fish challenged with *Aeromonas hydrophila*. Fish performance (weight gain and feed conversion) was improved in groups supplemented with omega-3-NPs (2 and 3 g/kg diet). The deposition of omega-3-PUFAs in fish flesh elevated with increasing dietary omega-3-NPs. Simultaneously the oxidative markers (H_2_O_2_, MDA, and reactive oxygen species) in fish flesh were reduced, especially with higher omega-3-NPs. Post-challenge, downregulation of IL-1β, IL-6, IL-8, TNF-α, and caspase-1 were noticed after dietary supplementation of omega-3-NPs. Moreover, mRNA expression of autophagy-related genes was upregulated while the mTOR gene was downregulated with higher omega-3 NPs levels. Lower expression of *A. hydrophila* ahyI and ahyR genes were detected with omega-3 NPs supplementation. In conclusion, omega-3-NPs application can fortify tilapia flesh with omega-3-PUFAs and augment its performance, immunity, and disease resistance against *Aeromonas hydrophila*.

## 1. Introduction

Farmed fish reared under an intensive system are exposed to high stocking density, short-term food deprivation in response to overproduction and disease outbreaks that stress the fish immune system and cause great losses in the aquaculture industry [1,2]. The interplay between the quality of nutrients consumed by fish and the immune system is among the most targets in the modern intensified sector that is intended for maintaining optimal growth rate and providing protection against different pathogens [3,4,5]. Nowadays, human consumers often perceive the consumption of tilapia flesh with high nutritional value owing to their health benefits [6]. The poly-unsaturated fatty acid (PUFAs) contents, particularly the omega PUFAs (especially omega-6 and -3), have become an important nutritional element in relation to human health. Additionally, both types of omega are required for fish and humans; however, the *n* − 6:*n* − 3 ratio is a very important topic as with increasing the proportion of *n* − 6 than *n* − 3 PUFAs, the risk of harmful effects, including inflammation, impairment of mental health and incidence of cardiovascular disease will be increased [4,7]. Fish is a rich source of omega-3, especially eicosapentaenoic acid (EPA) and docosahexaenoic acid (DHA), which are known to have numerous health benefits for consumers, however farmed fish as tilapia are increasingly exposed to dietary plant sources, rich sources in omega-6, often without considering their risk on fish health [8]. The formulated diet is the primary factor impacting the *n* − 6 and *n* − 3 FA ratios in tilapia, and supplementation of omega-3 PUFAs in the Nile tilapia diet can alter the body composition and augment its health [9]. On the other hand, during oxidative stress, antioxidants are capable of reducing cell damage by scavenging dangerous free radicals and providing protection against pathogens [10]. Physiological modifications are triggered by disease conditions as a result of an imbalance between reactive oxygen species (ROS) and nitrogen species generation and immune responses [11]. Additionally, *Aeromonas* septicemia is one of the dangerous diseases facing fish that is caused by *Aeromonas hydrophila* (*A. hydrophila*), particularly those are under a wide variety of stressful environmental conditions [12]. For counteracting the fish immune response, pathogenic *A. hydrophila* develops many mechanisms for spreading infections. Moreover, its pathogenicity depends on a variety of virulence factors, including proteases, motility, hemolysins, adhesions, toxins, and biofilm formation [13]. Various findings have displayed that dietary EPA and DHA can substantially alter fish-specific and nonspecific immunity and its capacity to fight infection [14] other than immune-related gene expression [15]. Because fish’s ability to resist pathogens and deal with stress differs to a large degree on their nutritional condition; thus, the need for a novel dietary formulation that would improve fish health and support sustainable aquaculture future industry has become an urgent concern. The efficient delivery of omega-3-PUFAs in the fish diet is facing major technical challenges owing to their nature of high unsaturation, which results in deterioration in lipids properties [16]. Nanotechnology has wide-ranging functions in aquaculture and could drastically assist in transforming this industry [17]. It has several functions, including encapsulation, enhanced bioavailability [18], and monitoring the release of functional compounds [19]. Incorporation of essential nutrients into nanoparticles (NPs) with unique physicochemical properties aimed at enhancing their bioactivity and bioavailability [20,21]. Nanostructured systems are a hopeful alternative for safeguarding omega fatty acids from oxidation by enhancing stability and solubility, thus sustaining food products with their sensory characteristics and nutritional value [22]. In this context, omega-3 nano-formulation can display innovative properties that augment its bioavailability and functional properties and, consequently, support optimal growth and health of farmed Nile tilapia. To the best of our knowledge, there are no data contemplating the effectiveness of omega-3-loaded nanoparticles on the fatty acid composition, antioxidant indicators and disease resistance of Nile tilapia. Thus, our study was directed to address the preceding knowledge lack through fulfilling the role of omega-3-loaded nanoparticles on growth, molecular features monitoring parameters related to antioxidant and immune status together with its promising potential role on the expression of virulence genes of *Aeromonas hydrophila* challenging strain.

## 2. Materials and Methods

### 2.1. Ethical Statement

The experimental set of rules for animal was fulfilled in agreement with the Ethical Regulations of an Institutional Animal Care and Use Committee of Faculty of Veterinary Medicine (ZUIACUC/F/83/2021), Zagazig University, Egypt.

### 2.2. Formulation and Characterization of Omega-3 Nanoparticles

**Figure 1:**
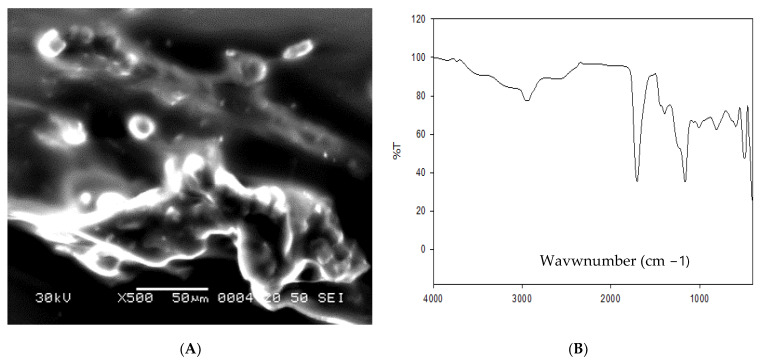
Scanning electron microscopy (**A**) and Fourier-transform infrared spectroscopy (FTIR, (**B**)) of omega-3 NPs.

For formulation of omega-3 nanoparticles, omega-3 (Natrol LLC 21411 Prairie Street Chatsworth USA), sodium alginate (A-2033), and acrylic acid (147,230) were used (Sigma-Aldrich, Saint Louis, MO, USA). Sodium alginate was liquified in distilled water (1.5%) using different concentrations of acrylic acid. In the next step, sodium alginate network structure was created by gamma irradiation utilizing solutions of hydrogels. In the next step, acrylic acid and sodium alginate solutions were mixed together until full compatibility. The previously made mixture was then exposed to gamma irradiation (40 kGy) at Atomic Energy Authority, Egypt. The characterization of formulated omega-3-NPs was performed via scanning electron microscope (Figure 1A) and Fourier-transform infrared spectroscopy (FTIR, Figure 1B).

### 2.3. Experimental Fish and Design

A total of 400 Nile tilapia fingerlings (*O. niloticus*) used in this study with an initial average body weight 14.14 ± 12 g was obtained from private fish farm, Abbassa village, Abu-Hammad district, Sharkia Governorate, Egypt. Fish were kept for two weeks for acclimatization before the experimental conditions then distributed into four experimental treatments in 20 glass tanks (20 fish/tank with five replicate treatment) filled with dechlorinated fresh water and aerated with central air compressor. The dissolved oxygen 5.7 ± 0.3 mg/L, water temperature ranged from 22.5−28 °C, pH was approximately 7.3 ± 0.1, while ammonium (NH4) and nitrite were found to be 0.20 ± 0.23 mg/L and 0.02 ± 0.05 mg/L respectively. The experimental treatments consisted of control group: fed control diet and other experimental treatments fed control diet supplied by graded levels of omega-3 long-chain poly-unsaturated fatty-acid-loaded nanoparticles (omega-3NPs) at the levels of 1, 2, and 3 g/kg diet.

### 2.4. Formulation of Experimental Diet

Nutrient compositions of experimental diets were formulated in response to Nile tilapia nutrient requirements described by Natural Research Council [23], as displayed in Table 1. The diet ingredients were thoroughly mixed, homogenized and pelleted then Omega-3 NPS was added to prepared feed mixture at different levels. Diets were then kept in a dark place at −20 °C to prevent Omega-3 NPS peroxidation until use. The nutrient composition (dry matter (DM), crude protein (CP), ether extract (EE), Ash, and crude fiber (CF)] of feed ingredients and diets were chemically analyzed via procedures described in [24]. The fatty acid composition of experimental diets is presented in Table 2. The nutritional trial continued for 12 weeks as fish were fed till apparent visual satiation three times daily. Feed residues remained at tank bottom and fish feces were drained off, and the tank’s water (around one quarter) was changed daily by clean fresh water.

### 2.5. Monitoring Nile Tilapia Performance 

Growth performance parameters of Nile tilapia after the feeding trial were calculated as described by Ibrahim, Nem, Ibrahim, Eissa, Fawzey, Mostafa, Abd El-Kader, Khater and Khater [18,25,26] as followings: Fish final body weight was evaluated to calculate final body weight gain (g) = Final mean body weight − Initial mean body weight. Weight gain percent = (Final average body weight − Initial average body weight)/Initial average body weight × 100. FCR = feed intake (g)/fish weight gain (g); specific growth rate, SGR% = (LN final bodyweight − LN initial body weight)/time (days) × 100; Feed intake, FI = feed consumed/number of fish per tank; Protein efficiency ratio, PER = weight gain (g)/protein consumed (g); Fulton’s condition factor (K) = fish weight (g)/length of fish (cm) 3 × 100 and survival rate (% ) = (fish number at the end of feeding trial/ fish at the beginning of experiment) × 100. Survival % = 100 × (final fish number/initial fish number).

### 2.6. Blood and Tissue Sampling 

At the end of 12 weeks, fish (five per replicate, 25 fish/treatment) used for sampling was anesthetized by submerging in Ethyl 3-aminobenzoate methanesulfonate suspension in 0.1 ppm (Sigma-Aldrich Chemie GmbH. Eschenstrasse., product No. E10521, Germany). Blood samples were taken from caudal blood vessels either with anticoagulant for blood picture or without anticoagulant into tubes for serum samples collection. The collected blood without anticoagulant was centrifuged at (3000× *g* r.p.m. for 15 min) for separation of sera. The serum samples were collected and then deposited immediately in deep freezer (−20 °C) until utilized for biochemical and immunological tests. Additionally, fish flesh samples were freeze-dried to analyze fatty acid contents and oxidative stress-related biomarkers. Muscle, liver, and splenic samples were collected immediately, and intestinal tissues were rinsed with normal saline solution and kept in liquid nitrogen for additional analysis of gene expression. 

### 2.7. Fatty Acid Profile

Fat was extracted from fish flesh samples as described in the previous procedure by [27]. Briefly, 0.5 g of collected samples were thoroughly mixed with of methanol (5 mL), water (0.4 mL) and chloroform (2.5 mL) with applying automatic shaking for 60 min. For further initiating a biphasic system, chloroform and Na_2_SO_4_ (1.5% solution) were added by 2.5 mL per each, and then the mix was centrifuged for 5 min at 1600× *g*. Fatty acid methyl esters were prepared by mixing hexane and methanolic solution. Finally, analysis of fatty acid was assessed by gas chromatography (model 3400CX, Varian, Palo Alto, CA, UAS), with high-quality capillary columns and ezchrom integration method, carrier gas (He); split 100 mL/min. Oven temperature 200 °C injector and detector 250 °C.

### 2.8. Oxidative Stress-Related Biomarkers in Muscle

Fish flesh homogenate (1:10 *w*/*v*) was prepared with Tris- HCl buffer and centrifuged for at 2000× *g* for 15 min; then, the collected supernatants were stored at −80 °C till further evaluation. Oxidation method was utilized to assess the contents of reactive oxygen species (ROS) in fish flesh [28]. The levels of hydrogen peroxide (H_2_O_2,_ μmoL/g of tissue) in fish flesh were estimated corresponding to the techniques detailed formerly by Loreto and Velikova [29]. Malondialdehyde (MDA) were analyzed utilizing the thiobarbituric acid reaction as previously identified by Livingstone et al. [30]. The total antioxidant capacity (T-AOC) was measured via equivalent diagnostic kits (Nanjing Jiancheng Bioengineering Institute, Nanjing, China) as described by the company’s guidelines.

### 2.9. Hematological and Serum Biochemical Evaluation

Red blood cells (RBCs) were estimated via a Neubauer hemocytometer (Sigma-Aldrich, Germany) and Natt-Herrick solution, and hemoglobin (Hb) levels were assessed by the use of the cyanomethemoglobin colorimeteric technique. Hematocrit (Ht) was quantified in the partial heparinized entire blood using the microhematocrit tube method [31]. Serum albumin, total protein, triacylglycerol (TAG), total cholesterol (TC), LDL-cholesterol, HDL-cholesterol, and VLDL-cholesterol, creatinine urea AST (aspartate amino transferase) and ALT (alanine amino transferase), were evaluated by a commercially accessible analytical kit (Spinreact, S.A./S.A.U. Ctra. Santa Coloma, 7 E-17176 Sant Esteve De Bas, Spain). 

### 2.10. Immunological Parameters Assessment

Immunoglobulin M (IgM) was defined using 96 test ELISA Kit. Catalog No. CSB-E12065Fh. Cusabio Biotech CO., Ltd (Houston, TX, USA). according to [32]. The assay of Agarose gel cell lysis was used for serum lysozymes activity detection, comparable to the approach outlined by [33]. The activity of myeloperoxidase (MPO) was anticipated following the formerly described procedure by Yilmaz, et al. [34]. Alternative complement activity was assessed according to Andani et al. [35].

### 2.11. Quantitative Real-Time PCR

Muscle, intestinal and splenic and liver samples for detecting the expression of antioxidant-associated genes; [*SOD* (superoxide dismutase), *CAT* (catalase) and *GPX* (glutathione peroxidase)], immune-related genes; [*IL-1β* (interleukin-1*β*), *IL-8* (interleukin-8), *IL-10* (interleukin-10), and *TNF-α* (tumor necrosis factor-α)], mechanistic target of rapamycin (*mTOR*), caspase-1, microtubule-associated proteins 1A/1B light chain (LC3) and Beclin-1 (BCLN-1) and autophagy-related genes (atg5 and atg12),. The gene sequence of the primers used is presented in Table 3. Total RNA was extracted using QIAamp RNeasy Mini Kit (Qiagen, Hilden, Germany) in accordance with the manufacturer’s instructions. Quantity and purity of extracted RNA were determined using Nano-Drop^®^ ND-1000 Spectrophotometer (NanoDrop Technologies, Wilmington, NC, USA). The total RNA was reverse transcribed using a QuantiTect Reverse Transcription Kit (Qiagen, Germany) as described by the producer’s instructions. The quantization of selected gene expression was measured by quantitative reverse transcription PCR (qRT-PCR) by QuantiTect SYBR Green Kit (Qiagen, Hilden, Germany) with special primers for each gene. All reactions were achieved in triplicate in the real-time PCR device (Stratagene, La Jolla, CA, USA). The levels of mRNA expression of measured genes were standardized using β-actin as reference gene and estimated by Ct (2^−ΔΔCT^) method following to Livak and Schmittgen [36]. 

### 2.12. Aeromonas Hydrophila Challenge and Sampling 

A virulent *Aeromonas hydrophila* strain isolated from diseased Nile tilapia suffered from typical hemorrhagic septicemia outbreak was employed in the current study. Brain heart infusion broth (Oxoid, UK) was used for fortification for *Aeromonas hydrophila* strain at 37 °C for 24 h. Bacterial strain was fully identified Vitek^®^ 2 system (bioM’erieux, Hazelwood, MO, USA) and PCR assay utilizing primers targeting gyrB control gene according to instruction demonstrated in a former study [37]. According to proposed methods identified by [38], molecular identification of aer, hyl, lip, and ahp virulence genes *A. hydrophila* and antimicrobial susceptibility testing was used for identification of multidrug-resistant and virulent strain. Fish were tested to be free from free of *A. hydrophila* infection prior to challenge. Ten fish per tank were inoculated with 0.2 mL/fish of *A. hydrophila* culture intraperitoneally (1.5 × 10^8^ CFU/mL) at the end of feeding trial (12 weeks) [39]. For the preparation *of A. hydrophila* inoculum, the bacterial strain was regularly cultivated on tryptic soy broth (Oxoid, UK) at 37 °C for 24 h. Subsequently, the bacterial suspension was diluted in phosphate-buffered saline and accustomed to a concentration of 1.5 × 10^8^ CFU/mL., All challenged fish were maintained over daily close visual inspection for two weeks with examining mortality, clinical signs, and postmortem lesions. 

### 2.13. Expression Analysis of A. hydrophila Virulence Genes

At various time points (7 and 15 days post-challenge), five fish/treatments were used for the assortment of splenic tissues in order to investigate the effect of omega-3NPs on the *A. hydrophila* ahyI and ahyR virulence-related genes. In this context, 16 s rRNA was used as the housekeeping gene for rRT-PCR via the following primers sequences: 5′-GCACAAGCGGTGGAGCATGTGG-3′ (forward) and 5′-CGTGTGTAGC CCTGGTCGTA-3′ (reverse) [40]. Two primer pairs were employed to amplify ahyI and ahyR genes with the subsequent sequences: 5′-TCTGGAG CAGGACAGTTTCG-3′ (forward) and 5′-ATGATGCAGGTCAGTTCGCT-3′ (reverse) and 5′-TTTACGGGTGACCTGATTGAG-3 (forward) and 5′-CCTGGATGTCCAACTACATCTT-3 (reverse), respectively [40].

### 2.14. Statistical Analysis

All statistics were performed by SPSS v. 23.0 software (Chicago, IL, USA), and general linear model (GLM) was used for statistics analysis. For evulating the normality and the homogeneity of the variance among groups, Shapiro–Wilk’s and Levene’s tests were employed, correspondingly. Considerable differences among the mean values were tested by Tukey’s test. Data discrepancy in this work was stated by the means of standard error of means (SEM), and significance level was established at (*p*-value 0.05).

## 3. Results

### 3.1. Growth Performance 

General improvement in growth performance parameters was detected after dietary supplementation of omega-3 NPs for a 12-week feeding trial Table 4. Among dietary treatments, fish supplemented with a 2 and 3 g/kg diet of omega-3NPS had a significantly higher (*p* < 0.05) final body weight (96.56 and 95 g, respectively, and vs. 70.14 in the control group). The most prominent improvement (*p* < 0.05) of FCR, PER, SGR, and condition factor was detected in groups fed higher levels of omega-3NPS. Meanwhile, no significant differences (*p* > 0.05) were noticed among different experimental groups regarding total feed intake.

### 3.2. Fatty Acid Composition and Antioxidant Biomarkers in Fish Flesh 

Table 5 describes the fatty acid composition of tilapia fish flesh after dietary supplementation of omega-3 NPs for 12 weeks. Notably, the fatty acid contents of fish flesh reflected that of the diet. It is worth noting that dietary supplementation of omega-3 NPs and EPA significantly increased sum PUFA and *n*-3 PUFA by increasing their levels in the diets in a dose-dependent manner. Moreover, the highest significant levels of DHA and EPA in fish flesh were detected in the group fed a 2 and 3 g/kg diet of omega-3 NPs. Interestingly, with increasing the levels of omega-3 NPs in Nile tilapia diets, the ratios of *n*-6/*n*-3 were significantly decreased (*p* < 0.05).

As shown in Table 5, H_2_O_2_ contents and ROS production in fish flesh were remarkably decreased (*p* < 0.05) with increasing dietary omega-3 NPs supplemental levels (*p* < 0.05). Furthermore, the most prominent reduction in MDA levels, lipid peroxidation markers, was noticed in the group fed a 3 g/kg diet of omega-3 NPs. In contrast, T-AOC activities were significantly elevated (*p* < 0.05) in groups supplemented with omega-3 NPs in a dosage-dependent manner.

### 3.3. Hematological and Serum Biochemical Indicators

Data relating to the hematological and biochemical interrelated indices are demonstrated in Table 6. Remarkably, no significant variations were noticed among all experimental groups regarding the RBC counts, Ht ratios and Hb concentrations. Concerning TP and globulins levels, they were significantly upraised in groups supplemented with a 2 and 3 g/kg diet of omega-3 NPs when compared with the control group (*p* < 0.05). Furthermore, no significant differences were found among all experimental groups regarding serum ALT, AST, urea, and creatinine variables. Additionally, the lipid profile showed that total cholesterol and LDL markedly declined in all groups supplemented with omega-3 NPs. Moreover, there was a drastic increase in HDL in all supplemented groups in a dose-dependent manner when compared with control. Meanwhile, no statistically significant (*p* < 0.05) differences were detected in TG and VLDL among the experimental groups.

### 3.4. Relative Expression of Antioxidant-Related Genes before Challenge in Muscle and Intestine

**Figure 2:**
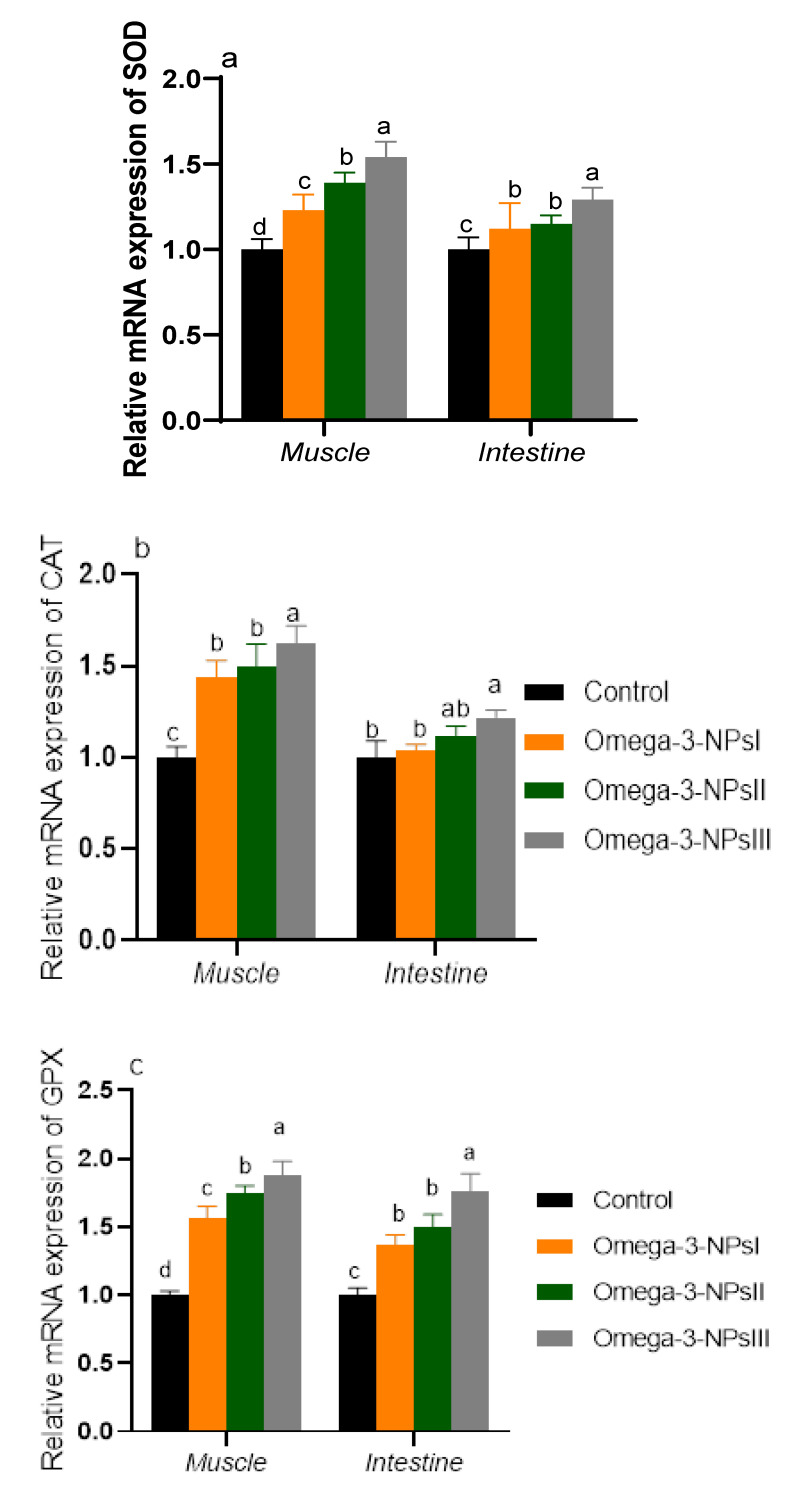
Effect of diets enriched with different supplemental levels of omega-3NPs (omega-3 nano particles) on real-time PCR analysis of oxidative-related genes [*SOD* (**a**), *CAT* (**b**), and *(GPX* (**c**)] expression in the muscle and intestine of Nile tilapia before challenge with *Aeromonas hydrophila*. Data are expressed as means ± SE. Bars with different letters denote significant differences (*p* < 0.05). Control: basal diet without omega-3-NPs omega-3-NPs I, II, and III: basal diet supplemented with omega-3-NPs at levels of 1, 2, and 3 mg/kg diet, respectively.

The mRNA expression of antioxidant-related genes is shown in Figure 2. Notably, the expression levels of antioxidant enzymes in muscles in is prominent than that of the intestine. Muscular SOD and GPX expression levels were upregulated in groups supplemented with omega-3 NPs in a dose-dependent manner. Moreover, the maximum expression levels of CAT were observed in the group fed omega-3 NPs at the level of 3 g/kg. Regarding intestinal GPX, SOD, and CAT, the most prominent upregulation was detected in the 3 g/kg omega-3 NPs supplemented group (increased by 1.76, 1.29, and 1.22, respectively, vs. the control group). 

### 3.5. Relative Expression of Immune-Related Genes Post-Challenge in Splenic Tissue 

**Figure 3:**
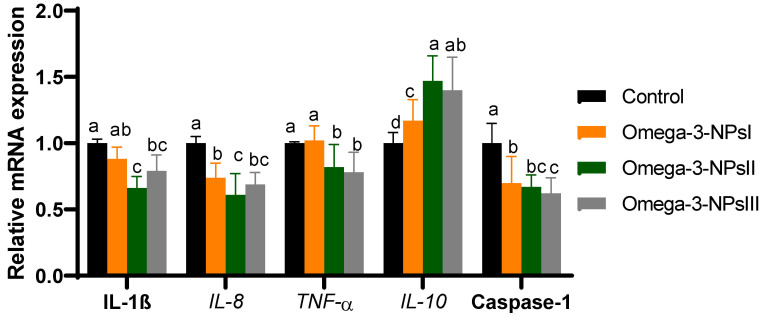
Effect of diets enriched with different supplemental levels of omega-3NPs (omega-3 nano particles) on real-time PCR analysis of immune-related genes [interleukin (*IL*)*-1β*, *IL-8*, *IL-10* and tumor necrosis factor α (*TNFα*) in Nile tilapia post-challenge with *Aeromonas hydrophila*. Data are expressed as means ± SE. Bars with different letters denote significant differences (*p* < 0.05). Control: basal diet without omega-3-NPs omega-3-NPsI, II, and III: basal diet supplemented with omega-3-NPs at levels of 1, 2, and 3 mg/kg diet, respectively.

The mRNA expression of splenic cytokine (*IL-1β*, *IL-6*, *TNF-α*, and *IL-10*) and caspase-1 were assessed post-challenge by *Aeromonas hydrophilia* Figure 3. The expression of IL-10 significantly upregulated (*p* < 0.05) with increasing levels of omega-3 NPs in a dose-dependent manner, and its maximum expression level was observed in the fish fed omega-3 NPs at the level of a 3 g/kg diet (increased to 1.4-fold). Feeding of Nile tilapia on a diet supplemented with higher levels of omega-3 NPs significantly downregulated (*p* < 0.05) the expression of *IL-1β*, *IL-6*, *IL-8*, and *TNFα* when compared with the challenged non-supplemented group. Regarding gene expression of caspase-1, feeding on omega-3 NPs downregulated its expression, especially with higher doses (2 and 3 g/kg diet; decreased to 0.67 and 0.62-fold) after *Aeromonas hydrophilia challenge* when compared to the control group.

### 3.6. Relative Expression of Autophagy-Related Genes Post-Challenge in Liver 

**Figure 4:**
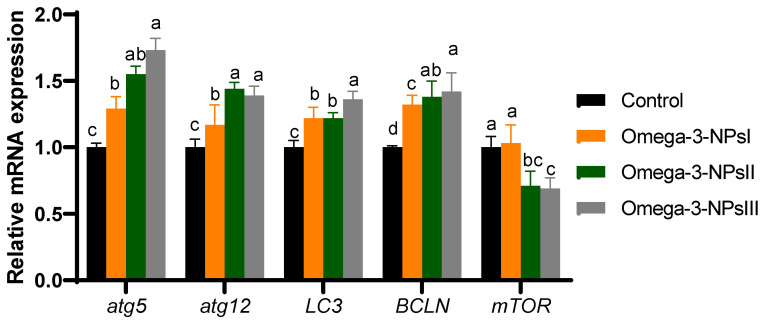
Effect of diets enriched with different supplemental levels of omega-3NPs (omega-3 nano particles) on real-time PCR analysis of autophagy-related genes [autophagy genes (atg5 and atg1microtubule-associated proteins 1A/1B light chain (LC3) and Beclin-1 (BCLN-1)), mechanistic target of rapamycin (mTOR)], and caspapase-1 in Nile tilapia post-challenge with *Aeromonas hydrophila*. Data are expressed as means ± SE. Bars with different letters denote significant differences (*p* < 0.05). Control: basal diet without omega-3-NPs omega-3-NPsI, II, and III: basal diet supplemented with omega-3-NPs at levels of 1, 2, and 3 mg/kg diet, respectively.

The effects of dietary omega-3 NPs supplementation on the relative gene expression of hepatic autophagy flux post-challenge with *Aeromonas hydrophilia* are presented in Figure 4. Compared to the challenged control group, the expression levels of *atg5* and *atg12* in the liver of omega-3 NPs supplemented groups were elevated substantially (*p* < 0.05), especially at higher levels (2 and 3 g/kg diet). However, the highest expression levels (*p* < 0.05) of *Lc3*-II were found in the group fed a 3 g/kg diet of omega-3-NPs. In addition, the expression levels of *Beclin1* were upregulated maximally in omega-3-NPs at the levels of 2 and 3 g/kg diet from omega-3 NPs supplementation. On the contra, the expression level of *mTOR* was remarkably lower in the higher levels of omega-3 NPs supplemented groups, unlike the control group.

### 3.7. Effect of Omega-3 NPs on Relative Expression of A. hydrophila Virulence-Related Genes and Survival Percentage

**Figure 5:**
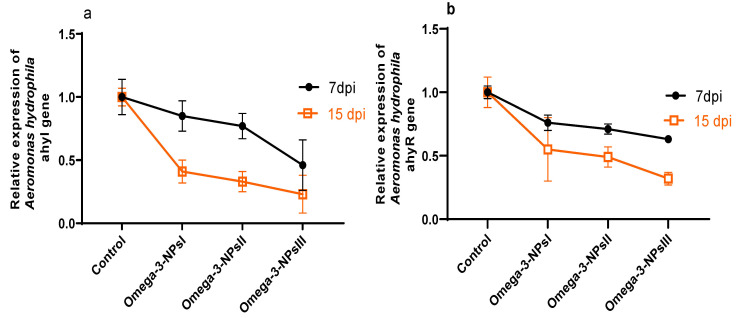
Effect of diets enriched with different supplemental levels of omega-3NPs (omega-3 nano particles) on relative mRNA expression levels of *Aeromonas hydrophila* virulence genes; and ahyR (**a**) and ahyI (**b**) 7 and 15 days post-challenge. Data are presented as means ± SE. Different letters indicate a statistical significance (*p* < 0.05). dpi: days post-challenge. Control: basal diet without omega-3-NPs omega-3-NPsI, II, and III: basal diet supplemented with omega-3-NPs at levels of 1, 2, and 3 mg/kg diet, respectively.

**Figure 6:**
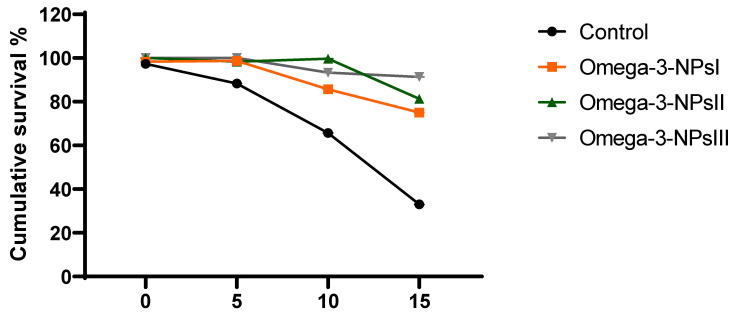
Effect of diets enriched with different supplemental levels of omega-3NPs (omega-3 nano particles) on cumulative survival percent in Nile tilapia after challenge with *Aeromonas hydrophila*. Control: basal diet without omega-3-NPs omega-3-NPsI, II, and III: basal diet supplemented with omega-3-NPs at levels of 1, 2, and 3 mg/kg diet, respectively.

The effect of omega-3 NPs on the expression of *A. hydrophila* virulence genes encoding ahyI and ahyR is presented in Figure 5. The data of real-time quantitative PCR revealed that omega-3-NPs significantly downregulated (*p* < 0.05) the expression of the ahyI and ahyR virulence genes 7- and 15-days post-challenge when compared to the control group. It was also noted feeding on a 3 g/kg diet of omega-3-NPs had the most depressing effect on such genes, especially at 15 days post-challenge. The cumulative survival percent ranged from 33 to 91%, where the highest survival percent post-challenge was detected in the group fed a 3 g/kg diet of omega-3-NPs Figure 6.

## 4. Discussion

Consumption of a diet enriched with omega-3 (*n* − 3) PUFAs, especially EPA and DHA, can promote an anti-inflammatory environment within the fish body and thereby strengthening its resistance to infectious diseases and targeting maximum production [41]. Additionally, the health importance of *n* − 3 fatty acids to humans is the anticipation of cardiovascular diseases, enhancement of visual insight, and strengthening of mental wellbeing [42]. With increasing global interest in changing dietary lifestyles, the production of Nile tilapia fillets that are enriched with long-chain poly-unsaturated fatty acids (LC-PUFAs) can be achieved by enriching their diets with such valuable functional nutrients. However, multiple double bonds in the structure of *n*-3-PUFAs make them labial for oxidation with inferior quality. Thus, protecting the delivery of *n*-3-PUFAs by their incorporation into nanoparticles can protect them from deterioration, improve their bioavailability and enhance their bioactivity. In the current study, incorporation of *n*-3-PUFAs into nanoparticles supports their beneficial role as evidenced by the enhanced growth rate, immune modulation that augmented their protecting role against diseases, and successful enrichment of fish fillet by *n*-3-PUFAs. Notably, the fish weight gain and FCR were enhanced after dietary supplementation omega-3-NPs even at lower levels compared with previous research [9]. Similarly, our results were congruent with [9], which reported improved specific growth rate, protein efficiency, daily growth index, relative feed intake, and reduced FCR in *P. hypophthalmus* with high supplementation by dietary DHA and EPA fatty acids. In the same line, Xu, et al. [43] reported improved growth performance of fish when supplied with DHA and EPA with decreasing ratio between omega-6: omega-3 fatty acids in the fish ration. The improved performance of fish indicated that EPA and DHA had a role in fish growth and metabolic regulation that resulted from the stimulation of insulin-like growth factor-1 and the Akt-mTOR-p70S6K pathway [44]. Moreover, it was proven that improved growth performance due to omega-3 supplementation might be linked with the improved health condition to the intensive systems conditions of fish production; fish were more subjected to disease challenges that increase the excretion of immune-related compounds such as acute-phase proteins and cytokines [45]. Additionally, the increased release of proinflammatory cytokines is the main cause of reducing feed intake and growth performance and deteriorating feed efficiency [44]. In the same line, we supposed that supplementation of Nile tilapia with long-chain omega-3 fatty acid (EPA, DPA, and DHA) in nano form may increase its absorption and protect them from peroxidation that maximizing their beneficial effect on the fish’s body.

Deposition of certain types of fatty acids in tilapia liver and filets is directly affected by supplementation of these acids in their diet, especially long-chain PUFAs, when fixing the environmental factors affecting fatty acids metabolism [7]. Copious trials have found a slight increase in long-chain PUFAs in tilapia flesh after dietary supplementation with linseed oil as a rich source of ALA(18:3 *n* − 3) [46,47], and this can be attributed to the limited ability of Nile tilapia to elongate and de-saturate (alpha linolenic acid, ALA) into long-chain PUFAs (EPA, DPA, and DHA) [48]. The efficacy of the human body to convert ALA into longer-chain omega-3 fatty acids (EPA, DPA, and DHA) is limited (less than 10%), so it is crucial to be supplied by long-chain omega-3 fatty acids in the food [49,50]. In this regard, supplementation of EPA and DHA in the Nile tilapia diet was considered as a prospective approach that ensures their deposition in fish flesh with desired levels that fulfilling consumers’ demands. In the current study, increasing levels of EPA and DHA in Nile tilapia diets elevated their levels in fish flesh. In agreement with our results, Stoneham et al. [7] reported increased liver and filet levels of long-chain PUFAs with supplementation with fish oil that is rich in long-chain PUFAs. Additionally, the inclusion of *Schizochytrium* sp. in tilapia feed, which is rich in omega-3 and DHA fatty acids, altered the lipid profile and fatty acids composition, especially long-chain PUFAs in the fish’s body, as it increased linearly with increased supplementation level [51]. Moreover, supplementation of algae enriched with DHA increased its deposition in the muscle of sea bream (*Sparidentex hasta*) [52]. On the other hand, the protection of omega-3 from peroxidation is an important issue in increasing their bioactivity inside the fish’s body. In this respect, our results revealed that incorporation of omega-3 in a nano form was associated with their higher deposition in fish flesh, indicating the efficacy of the nano delivery system for omega-3 to fish fillet. Herein, the contents of total cholesterol and LDL-C in the serum of the fish group fed omega-3-NPs were prominently reduced than that in the control group. Meanwhile, HDL-C levels showed a contrasting trend in this group which is in agreement with Peng, et al. [53]. Similarly, *n* − 3 LC-PUFA can inhibit LDL-C and VLDL uptake and degradation [53]. Moreover, dietary supplementation of omega-3 reduced levels of cholesterol, triglyceride, and VLDL [9].

Stressful conditions that fish face under an intensive culture system predispose them to many associated health problems that affect their growth rate. Thus, strengthening fish antioxidant systems to cope with oxidative stress via dietary modulation is an urgent issue. Fish health and immunity are strongly connected to the antioxidant defense system. Under usual physiological circumstances, fish continuously create free radicals and are constantly cleared, and their concentration is kept under a dynamic equilibrium [54]. When fish are exposed to stress, they unavoidably generate elevated levels of ROS that may result in considerable cell damage. Additionally, higher ROS levels can initiate cell membrane lipids peroxidation that impairs fish performance and health. CAT, SOD, and GSH-PX are the main enzymes that protect the cells against reactive oxygen species (ROS) and are also considered the first line of defense, which catalyzes the devastation of hydrogen and lipid peroxidation over GSH as an electron contributor [55]. In this regard, lowering fish flesh ROS and H_2_O_2_ levels after supplementation of omega-3 NPs imply reduced free radical contents and lipid damage. This can be explained by the beneficial role of dietary omega-3 NPs that can depress ROS generation [9] via enhanced cellular proficiency toward oxidative stress. Furthermore, the capacity of omega-3 NPs to remove H_2_O_2_ from fish flesh was confirmed by lowering its concentrations in fish fillets in all omega-3 NPs supplemented groups contradictory to the control group. It has been shown that MDA is a product of lipid peroxidation and can be used as a marker to determine the degree of damage resulting from oxygen-free radicals inside body cells [56]. Herein, the contents of MDA in fish flesh decreased with increasing dietary omega-3 PUFA levels. This can be explained by the protection of dietary omega-3 PUFAs from oxidation via their incorporation in nano form. Additionally, CAT, SOD, and GPx are the important key enzymes for the regulation and preservation of anti-oxidative status in humans and animals, including fish. In our study, the supplementation of EPA + DHA enhanced the anti-oxidative status, as detected by their higher levels in muscle and intestine before the challenge when compared with the control group. In agreement, Zuo et al. [14] also reported that SOD activities are boosted by increasing the dietary levels of omega-3 PUFAs from 0.15% to 0.60%.

Fish immune systems confer protecting them from invasive pathogenic microbes such as viruses or bacteria. Strengthening of the fish immune system by dietary poly-unsaturated fatty acids (PUFAs) with a special focus on omega-3 PUFAs α-linolenic acid (ALA), eicosapentaenoic acid (EPA), and docosahexaenoic acid (DHA) greatly associated with their immune-regulatory properties [57]. It has been shown that dietary fatty acid composition prompted nonspecific immunity (e.g., serum lysozyme, phagocytosis, respiratory burst), specific immunity (e.g., antibody production and resistance to pathogens), eicosanoid production, and immune-related genes expression [14,58,59] in fish. To the best of our knowledge, the mechanisms implicated in the modulation of fish immune systems by modifying the expression of immune and autophagy-related genes by omega-3 nano form have not yet been considered. Lysozymes are vital defense antimicrobial proteins of the immune system that is associated with the first barrier of innate immunity in fish and have lysis activity against pathogenic bacteria [16,17]. Additionally, immunoglobulin has a very important role in defense mechanisms via killing microbes and pathogens, restricting the spread of infectious agents, and repairing maintenance tissues [18]. In the current study, our results described that using various levels of omega-3 NPs enhanced the fish immune system before challenge (total protein, lysozymes, myeloperoxidase, and immunoglobulin M). Moreover, supplementation of omega-3 NPs, even at lower levels, drive immune responses into an anti-inflammatory direction as proved by downregulation of proinflammatory cytokine gene expression with upregulation of anti-inflammatory *IL-10* gene expression in Nile tilapia post-challenge with *Aeromonas hydrophila*. These results indicated that using omega-3 NPs could improve the immune response of Nile tilapia. In accordance, supplementing of dietary *n* − 3 PUFA of increased the activity of Lysozymes and complement before infection [58]. Herein, post-challenged, the expression of *IL-10* was upregulated while the expression of *IL-1β* was downregulated in the group fed a 3 g/kg diet of omega-3 NPs than other groups, which is in agreement with An et al., [60]. Moreover, in sea bream, the intestinal *TNF*-α and *IL*-*1β* expression were higher even at lower LC-PUFA supplementation levels after the injection of *Photobacterium damselae* subsp. *piscicida* [15]. Omega-3 fatty acids have the capability to lessen inflammation via reducing the manufacture and emission of cytokines and chemokines by macrophages [61]. Recently, [15] proved the beneficial role of dietary omega-3 enriched oils in modulating the expression of cytokines-related genes against mixovirus (Mx protein) in marine fish. Moreover, dietary feeding on omega-3 fatty acids improved the immunity of the fish as detected by increasing myeloperoxidase and total immunoglobulin levels [9] and these positive affect may be related to increasing dietary levels of omega-3-NPS that reduces the synthesis of omega-6-derived metabolites which promotes inflammation [62]. Feeding of sea bream (*Sparidentex hasta*) on supplemental DHA could enhance the serum immunity parameters such as lysozyme and phagocytic activity of fish and modulate the expression of (*IL*-*1B*, *IL-6*, and *IL-10*) [52]. Furthermore, DHA exerted potent inhibitory effects on proinflammatory cytokines expression in large yellow croakers [63]. Notably, the incorporation of omega-3 into nano-formulation boosted their immune-modulatory properties in our study, even in small doses owing to increasing their bioavailability and delivery to target tissues and protecting them from degradation, which is in accordance with [64]. Autophagy is a process in which cells utilize lysosomes to break down their damaged macromolecular and other organelles [65]. Autophagy and innate immunity may operate together via a combined signaling pathway [66,67]. The immune system utilizes the degradation products of autophagic cytoplasmic material to control adaptive immunity [68]. Accumulating evidence revealed that the ratio between *n* − 6/*n* − 3 PUFAs can not only provoke cell autophagy but also influence the host ability to avoid autophagy of intracellular pathogen [69,70]. There is no study described the role of omega-3-NPs on autophagy-related genes post-challenge in fish. Herein, the other interesting finding after challenge was that even moderate levels of omega-3NPs had been capable of inducing the expression of autophagy-related genes (*atg12*, *atg5*, *LC3-II*, and *BECN1* in the liver) and thereby indicated faster clearance of *Aeromonas hydrophila*. The incidence of autophagy depends on the involvement of a unique series of autophagy-related proteins as *atg12*-*atg5* and these proteins has a crucial role in the induction, nucleation, expansion, termination, maturation, and autophagosomes degradation [71]. The autophagy protein atg5, which is fairly well preserved across most eukaryotes, has a vital role in the autophagic vacuoles development [72]. The abundance of LC3 is considered as a marker of autophagy [73]; therefore, the increase in LC3 is linearly correlated to the number of autophagic vacuoles. In our study, the mRNA expression of *LC3*-II was prominently upregulated in the group supplemented with a 3 g/kg omega-3NPspost-challenge compared to the control group. The BECN1 gene is considered a homolog of yeast atg6 and a target gene engaged in autophagy in mammalian cells. Moreover, *BECN1* is not only concerned in the autophagosomes formation, but also has an essential role in tumor formation by controlling autophagic activity [74]. In the present study, *BECN1* expression was significantly upregulated in the liver in the high omega-3NPs supplemented group post infection more than challenged-non-supplemented group indicated their role in initiating autophagy-related genes. Additionally, mTOR, a serine/threonine kinase, is a key role in cell metabolism and plays a critical role in the pathway autophagy [75] as there is a reverse relation to autophagy initiation and the activation of mTOR [75]. Herein, mTOR mRNA expression was downregulated in groups supplemented with omega-3 NPs post-infection, which is in line with induction of autophagy. In accordance, DHA decreased the phospho-Akt and phospho-mTOR levels in a dose-related manner and thus provoked autophagy [76]. Moreover, the mode of action of DHA is decreasing inflammasome stimulation by enhancing autophagy and thus reducing IL-1β secretion [77]. Nutritional immunology is a new approach to disease prevention in aquaculture via using nutrients to overcome drug residues and resistance and vaccination program difficulties [78,79]. Previous findings described n-3 PUFA as a crucial nutrient that regulates the immune system of fish in response to pathogen invasion [15,80,81], but there is no information describing the efficacy of omega-3NPs against the invasion of *Aeromonas hydrophila*. Interestingly, groups supplemented with moderate levels of omega-3NPs showed a lower cumulative mortality rate in accordance with remarkable downregulation of ahyI and ahrR virulence genes of *Aeromonas hydrophila*. These outcomes revealed for the first time that DHA and EPA modules of omega-3 have anti-virulent properties against *Aeromonas hydrophila*. Similarly, dietary feeding on omega-3 fatty acids reduced the infection against *Aeromonas hydrophila* in *P. hypophthalmus* [9]. In accordance, the highest proinflammatory responses caused by *Aeromonas hydrophila* in the challenged group were reduced in groups supplemented with omega-3 NPS, especially at the higher levels. Dietary n-3 PUFA may regulate fish immunity and disease resistance against parasites (*Cryptocaryon irritans*) [14]. Additionally, Dietary n-3 unsaturated fatty inhibits the inflammatory response of hybrid grouper and provides protection against Vibrio harveyi [58]. Moreover, higher levels of EPA and DHA could inhibit bacterial growth and boost the secretion of anti-inflammatory cytokines, in this manner protecting zebrafish from *V. vulnificus* disease [78]. The antibacterial effect of omega-3 FAs was explained by their anti-virulence properties, which change cell membrane hydrophobicity, alter the charge on the cell surface, and change membrane integrity, which leads to electron outflow and subsequent cell death [79].

## 5. Conclusions

Using a nanocarrier-based system was proven to enhance the bioactivity and stability of delivered bioactive omega-3 PUFAs. Notably, the enhanced mode of action of omega-3-NPs, even in moderate concentrations, can be explained by its protection from environmental conditions till reaching target tissues. This study indicated for the first time that the application of omega-3-PUFAs containing nano-formulations could improve the growth performance of Nile tilapia and enrich its flesh with a sufficient amount of EPA and DHA that can reach human consumers. Additionally, the nano-formulated form of omega-3 PUFAs can modulate Nile tilapia immune system, induce autophagy-related genes and switch off certain genes that are responsible for the virulence of Aeromonas hydrophilia, thus hindering its infection. Finally, it is recommended that using omega-3-NPs even at moderate levels can maximize Nile tilapia performance and strengthen its immune system against pathogenic microbes.

## Figures and Tables

**Table 1:** Ingredients and chemical composition of the basal diet.

Ingredients, %	Control Diet	Supplemental Omega-3-NPs (g/kg Diet)		
		0.05%	0.1%	0.2%
Fish meal	16.5	16.5	16.5	16.5
Soybean meal	33.2	33.2	33.2	33.2
Yellow corn	28	28	28	28
Corn gluten	4	4	4	4
Rice bran	12	12	12	12
Soy oil	3.5	3.45	3.4	3.3
Lysine	0.10	0.10	0.10	0.10
DL- Methionine (98%)	0.20	0.20	0.20	0.20
Threonine	0.10	0.10	0.10	0.10
Di-calcium phosphate	1.20	1.20	1.20	1.20
* Vitamins and minerals premix	1.20	1.20	1.20	1.20
DHA + EPA	0	0.05	0.1	0.2
Chemical analysis				
Digestible energy (kcal/kg)	2926	2922	2918	2909
Crude protein, %	32.10	32.10	32.10	32.10
Ether extract, %	4.71	4.71	4.71	4.71
Ca, %	0.80	0.80	0.80	0.80
Available P, %	0.43	0.43	0.43	0.43
Lysine, %	2.00	2.00	2.00	2.00
Methionine, %	0.81	0.81	0.81	0.81

* Vitamins and minerals mixtures/kg of product: 130 mg biotin, 4500 mg pantothenic acid, 32 mg cobalt, 180 mg folic acid, 2200 mg copper, 60 mg selenium, 0.75 g antioxidant, 17,200 mg zinc, 200 mg iodine, 4000 mg manganese, 810 mg iron, 3000 mg niacin, 1250 mg vitamin B1, 2600 mg vitamin B6, 2600 mg vitamin B2, 3750 L mg vitamin, B12, 32,000 mg vitamin C, 1,000,000 IU vitamin A 300,000 IU vitamin D3, 30,000 IU vitamin E and 600 mg vitamin K.

**Table 2:** The composition of fatty acid of the experimental diets (%).

Fatty Acids	Supplemental Omega-3-NPs (g/kg Diet)			
	Control	1	2	3
18:2*n* − 6	2.053	2.053	2.053	2.053
C18:3*n* − 6 (Gamma linolenic acid)	0.002	0.002	0.002	0.002
C18:3*n* − 3 (Alpha linolenic acid)	0.074	0.074	0.074	0.074
C18:4*n*3 (Stearidonic acid)	0.000	0.08	0.01	0.000
20:2*n* − 6	0.07	0.00	0.00	0.000
20:3*n* − 3	0.06	0.000	0.00	0.07
20.4 *n* − 6	0.004	0.000	0.000	0.06
20:5*n* − 3 EPA	0.201	2.01	3.81	7.41
22:2 *n* − 6	0.008	0.006	0.03	0.002
C22:5*n* − 3 Docosapentaenoic acid (DPA)	0.037	0.037	0.037	0.037
C22:6*n* − 3 Docosahexaenoic acid (DHA)	0.095	1.30	2.50	4.90
∑*n* − 3 PUFA	0.41	1.41	2.41	3.42
∑*n* − 6 PUFA	2.01	2.01	2.01	2.01
*n* − 6/*n* − 3	5.03	1.43	0.83	0.59

∑*n* − 6 PUFAs: sum of *n* − 6 poly-unsaturated fatty acids. ∑*n* − 3 PUFAs: sum of *n* − 3 poly-unsaturated fatty acids.

**Table 3:** Primers sequence employed for analysis of quantitative real-time PCR.

Gene	Sequence	Accession No.
*SOD*	F-GACGTGACAACACAGGTTGC R-TACAGCCACCGTAACAGCAG	XM_003449940.5
*CAT*	F-TCAGCACAGAAGACACAGACA R-GACCATTCCTCCACTCCAGAT	XM_031754288.1
*GSH-Px*	F-CCAAGAGAACTGCAAGAACGA R-CAGGACACGTCATTCCTACAC	NM_001279711.1
*IL-1β*	F-TGCTGAGCACAGAATTCCAG R-GCTGTGGAGAAGAACCAAGC	XM_019365841.2
*IL-6*		
*IL-8*	F-GCACTGCCGCTGCATTAAG R-GCAGTGGGAGTTGGGAAGAA	XM_031747075.1
*IL-10*	F-CTGCTAGATCAGTCCGTCGAA R-GCAGAACCGTGTCCAGGTAA	XM_013269189.3
*TNF-α*	F-GAGGTCGGCGTGCCAAGA R-TGGTTTCCGTCCACAGCGT	NM_001279533.1
*Caspase* 1	F: GCTGTCTGAGTAAGTGTATCAT R: CCAACACGTTAAAATGGATCTGA	XM_019367762.2
*Atg5*	F-ATTGGCGTTTTGTTTGATCTT R-TTTGAGTGCATCCGCCTCTTT	XM_019082404.1
*Atg12*	F-ACAGTACAGTCACTCGCTCA R-AAAACACTCGAAAAGCACACC	XM_019125508.1
*LC3-II*	F-GGAACAGCATCCAAGCAAGA R-TCAGAAATGGCGGTGGACA	NM199604.1
*BCLN1*	F-TCTGTTTGATATCATGTCTGG R-TAATTCTGGCACTCATTTTCT	XM_019068185.1
*mTOR*	F-TGCGGAGTATGTGGAGTT R-CATCTCTTTGGTCTCTCTCTGG	XM_019108641.1
*β-actine*	F-TGGCATCACACCTTCTATAACGA R-TGGCAGGAGTGTTGAAGGTCT	XM_003455949.2

*GSH-Px*: glutathione peroxidase, *SOD*: superoxide dismutase, *CAT:* catalase, *IL*: interleukin, *TNFα*: tumor necrosis factor *α*, *LC3-II:* Microtubule-associated proteins 1A/1B light chain, *BCLN1*: Beclin-1, *mTOR*: mechanistic target of rapamycin (*mTOR*).

**Table 4:** Growth performance parameters of Nile tilapia (*O. niloticus*) fed diets enriched with different levels of omega-3 NPs.

Parameter	Supplemental Omega-3-NPs (g/kg Diet)					
	Control	I	II	III	*p* Value	SEM
Initial body weight (g/fish)	14.02	14.26	14.14	14.14	0.216	0.029
Final body weight (g/fish)	70.14 ^c^	79.16 ^b^	96.56 ^a^	95.00 ^a^	<0.001	12.88
Final weight gain (g/fish)	56.12 ^c^	64.90 ^b^	82.42 ^a^	80.86 ^a^	<0.001	12.39
Final weight gain (%)	400.36 ^c^	455.21 ^b^	582.72 ^a^	571.72 ^a^	<0.001	526.22
Total feed intake (g/fish)	96.52	103.08	99.41	100.26	0.08	12.85
Feed conversion ratio	1.71 ^a^	1.58 ^b^	1.21^c^	1.24^c^	<0.001	0.002
Specific growth rate (%)	1.92 ^c^	2.04 ^b^	2.28 ^a^	2.27 ^a^	<0.001	0.002
Protein efficiency ratio	1.75 ^b^	1.89 ^b^	2.50 ^a^	2.43 ^a^	<0.001	0.016
Condition factor	2.31 ^c^	2.54 ^bc^	3.08 ^ab^	3.36 ^a^	<0.001	0.182

Mean values with different letters in the same row differ significantly at *p* < 0.05, QT-NPs: omega-NPs: omega-3 nanoparticles, SEM: standard error of the mean.

**Table 5:** Meat fatty acid analysis, lipid peroxidation and antioxidant biomarker of Nile tilapia (*O. niloticus*) fed diets enriched with various levels of omega-3-NPs.

Parameters	Supplemental Omega-3-NPs (g/kg Diet)					
	Control	I	II	III	*p* Value	SEM
ΣSFAs	17.23 ^a^	16.93 ^ab^	16.27 ^b^	15.2 ^c^	<0.02	0.25
ΣMUSFAs	25.23 ^a^	24.3 ^ab^	23.2 ^b^	22.1 ^c^	<0.001	0.39
Σ*n* − 3	5.9 ^d^	8.31 ^c^	11.39 ^b^	16.33 ^a^	<0.04	0.47
Σ*n* − 6	53.46 ^a^	45.36 ^b^	40.22 ^c^	35.69 ^d^	<0.02	0.29
ΣPUFAs	59.36 ^ab^	58.79 ^b^	61.315 ^a^	61.58 ^a^	<0.008	0.24
*n*6/*n*3	9.06 ^a^	5.45 ^b^	3.53 ^c^	2.19 ^d^	<0.001	0.13
MDA (nmol/g tissue)	21.30 ^a^	19.40 ^ab^	19.60 ^ab^	17.87 ^b^	0.045	1.40
ROS	112.80 ^a^	96.20 ^b^	67.23 ^c^	56.57 ^d^	<0.001	19.91
T-AOC (U/mg prot)	0.83 ^d^	1.62 ^c^	2.27 ^b^	3.73 ^a^	<0.001	0.03
H_2_O_2_ (μmoL/g tissue)	4.73 ^a^	3.33 ^b^	2.23 ^c^	1.69 ^c^	<0.001	0.09

SFAs (total saturated fatty acids), MUFAs (total monounsaturated fatty acids), PUFAs (poly-unsaturated fatty acids). ∑*n* − 6 PUFAs (total of *n* − 6 poly-unsaturated fatty acids). ∑*n* − 3 PUFAs = total of *n* − 3 poly-unsaturated fatty acids. MDA (malondialdehyde), ROS: (reactive oxygen species), T-AOC: (total antioxidant ability), H_2_O_2_ (hydrogen peroxide). Mean values with a variety of letters in the same row change significantly at *p* < 0.05, omega-NPs (omega-3 nanoparticles), SEM (standard error of the mean).

**Table 6:** Hematological and immunological indices and lipid peroxidation and antioxidant biomarkers of Nile tilapia (*O. niloticus*) fed diets supplemented with variable levels of omega-3-NPs for 12 weeks.

Parameters	Supplemental Omega-3-NPs (g/kg Diet)					
	Control	I	II	III	*p* Value	SEM
RBCs (×10^6^/μL)	2.32	2.59	2.52	2.56	0.580	0.07
Ht (%)	28.87	28.37	29.10	29.37	0.345	0.42
Hb (g/dL)	9.11	9.60	9.58	9.84	0.085	0.09
Total protein (g/dL)	3.07 ^c^	3.13 ^c^	4.20 ^b^	5.33^a^	<0.001	0.10
Albumin (g/dL)	1.99 ^b^	2.96 ^a^	2.38 ^ab^	2.28 ^b^	0.012	0.07
Globulin (g/dL)	1.07 ^d^	1.2 ^c^	1.71 ^b^	2.50 ^a^	<0.001	0.13
ALT (U/L)	47.17	48.37	45.23	47.20	0.578	7.23
AST(U/L)	29.71 ^ab^	27.71 ^b^	31.54 ^a^	28.54 ^ab^	0.041	1.87
Creatinine (mg/dL)	0.52	0.50	0.49	0.50	0.869	0.001
Urea (mg/dL)	5.00 ^a^	4.70 ^ab^	4.65 ^ab^	4.49 ^b^	0.054	0.04
Cholesterol (mg/dL)	92.53 ^a^	87.70 ^ab^	81.25 ^b^	69.74 ^c^	0.001	16.95
Triacylglycerol (mg/dL)	71.63	69.96	73.63	66.96	0. 241	13.92
HDL-cholesterol (mg/dL)	33.23 ^c^	42.66 ^b^	47.13 ^ab^	52.44 ^a^	<0.001	6.93
LDL-cholesterol (mg/dL)	44.98 ^a^	31.05 ^b^	19.39 ^b^	13.90 ^c^	<0.001	33.61
VLDL-cholesterol (mg/dL)	14.33	13.99	14.73	13.39	0.241	0.56
IgM (μg/mL)	24.90 ^c^	32.63 ^b^	36.84 ^ab^	38.74 ^a^	<0.001	6.00
Serum lysozyme (μg/mL)	0.76 ^c^	1.21 ^b^	1.37 ^b^	1.63 ^a^	<0.001	0.01
MPO (μmoL/L, OD 450 nm)	0.53 ^c^	0.63 ^c^	0.81 ^b^	1.07 ^a^	<0.001	0. 004
Serum alternative complementary (u/mL)	215.10 ^c^	228.33 ^b^	239.57 ^a^	250.29 ^a^	<0.001	24.18

RBCs (red blood cells), Ht, (hematocrit), Hb (hemoglobin), WBCs (white blood cells), ALT (alanine transaminase), AST (aspartate transaminase), HDL(high density lipoprotein), LDL (low density lipoprotein), VLDL (very low-density lipo-protein), IgM (immunoglobulin M), MPO (myeloperoxidase), Mean values with several letters in the same row diverge significantly at *p* < 0.05, omega-NPs (omega-3 nanoparticles), SEM (standard error of the mean). Serum lysozyme activity was substantially increased in the group supplemented with a 2 g/kg diet of omega-3 NPs followed by the group supplemented with 1 and 2 g/kg of omega-3 NPs compared to the control (*p* < 0.05) group. Furthermore, the highest values (*p* < 0.05) of alternative complement, myeloperoxidase, and immunoglobulin type M were detected in groups supplemented with a 2 and 3 g/kg diet of omega-3 NPs when compared with the control group.

## Data Availability

The data presented in this study are available upon request from the corresponding author.
